# Humoral Response to SARS-CoV-2 Vaccine of a Patient Receiving Methotrexate Treatment and Implications for the Need of Monitoring

**DOI:** 10.3390/vaccines9101151

**Published:** 2021-10-09

**Authors:** Krzysztof Lukaszuk, Izabela Woclawek-Potocka, Grzegorz Jakiel, Paulina Malinowska, Artur Wdowiak, Karolina Rozanska, Lukasz Rabalski

**Affiliations:** 1 Department of Obstetrics and Gynecology Nursing, Medical University of Gdansk, 80-211 Gdansk, Poland; krzysztof.lukaszuk@invicta.pl; 2 Invicta Research and Development Center, 81-740 Sopot, Poland; karolina.rozanska@invicta.pl; 3 Department of Gamete and Embryo Biology, Institute of Animal Reproduction and Food Research, Polish Academy of Sciences, 10-243 Olsztyn, Poland; i.woclawek-potocka@pan.olsztyn.pl; 4 The Center of Postgraduate Medical Education, 1st Department of Obstetrics and Gynecology, 01-004 Warsaw, Poland; grzegorz.jakiel1@o2.pl; 5 Diagnostic Techniques Unit, Medical University of Lublin, 20-081 Lublin, Poland; artur.wdowiak@umlub.com; 6 Laboratory of Recombinant Vaccines, Intercollegiate Faculty of Biotechnology, University of Gdansk and Medical University of Gdansk, 80-307 Gdansk, Poland

**Keywords:** COVID-19, vaccination, methotrexate, TNFis, autoimmune disease, AIIRD

## Abstract

We report a case of monitoring the antibody response to the BioNTech–Pfizer vaccine of a 50-year-old female diagnosed with rheumatoid arthritis undergoing treatment with methotrexate (MTX). Antibody levels were measured 21 days after dose 1 (i.e., on the day of dose 2) and then 8, 14 and 30 days after dose 2 with Elecsys Anti-SARS-CoV-2 S assay (Roche Diagnostics). Patient showed a negative result after dose 1 and had the serum sample retested using a LIAISON^®^ SARS-CoV-2 TrimericS IgG assay (DiaSorin), which showed a positive result. Subsequent samples were tested using both assays. Antibody levels kept increasing but at a much slower rate than in patients not receiving any immunomodulatory therapies. Other research indicates that among patients with autoimmune diseases, those receiving disease-modifying antirheumatic drugs (DMARDs) have higher COVID-19 mortality than those treated with tumor necrosis factor inhibitors (TNFis). These results indicate the need for people with autoimmune diseases to be carefully observed following vaccinations, including testing of antibody levels, and treated as potentially at risk until the effect of vaccination is confirmed. The different available vaccines should also be tested to verify their usefulness in the case of people with autoimmune diseases and those who take different immunomodulatory medications.

## 1. Introduction

The COVID-19 pandemic is moving to the prevention stage with mass vaccination. The rollout of vaccination programs has been slower than public expectations. It seems important to optimize the population-based vaccination system and to search for weak links in vaccination that may be subject to the illusion of safety despite the lack of response to vaccination.

So far, seven different vaccines have been approved for use in different regions of the world [1]. The approved and candidate vaccines are based on various platforms including mRNA, DNA, adenoviral vector-based, adjuvanted recombinant protein, and inactivated virus vaccines. It is worth noting that the differences go further than manufacturing technology. They have a different point of entry and can elicit different types of immune responses of varying strength. This seems to be important, especially for patients with autoimmune diseases in whom we deliberately disrupt their immune response as part of the treatment process. Some authorities have included patients receiving immunosuppressants in the high-risk group for developing severe COVID-19. However, at this point there is limited evidence regarding severity of COVID-19 and the response to vaccination in the group of autoimmune disease patients treated with methotrexate. This case report presents the case of a 50-year-old female diagnosed with rheumatoid arthritis (RA) treated with methotrexate (MTX) who received the BioNTech–Pfizer BNT162b2 COVID-19 mRNA vaccine (BNT162b2). The patient was monitored for antibody levels for 180 days after the second dose of the vaccine. The case patient’s results are compared to the group of healthcare workers who were also monitored for the same period of time, and additionally to the subgroup of females of a similar age. The report reviews the currently available literature concerning RA patients treated with MTX from the initially published reports covering the risks of COVID-19 infection [2,3] in that group and then the effects of vaccination [4,5,6,7].

## 2. Case Report

We report the case of a 50-year-old Caucasian female, BMI 27.7 kg/m^2^, with a 20-year history of seropositive rheumatoid arthritis (RA) and no other comorbidities. The patient has been treated with methotrexate (MTX) for 10 years. The most recent treatment cycle with MTX was started in May 2020. Prior to that, the patient underwent treatment with tocilizumab (RoActermra, Roche, Basel, Switzerland) starting in combination with MTX but later alone. The immediate effect of the biological treatment was very good but not lasting. Patient returned to MTX in May 2020 starting with a 30 mg/week dose. Adverse reactions prompted another break in MTX treatment and resumption from the lower dose in September 2020 starting with 15 mg/week, increased to 20 mg/week in November 2020 and to 25 mg/week in the second half of January 2021. During the vaccination follow-up period of 6 months, the dose remained set at 25 mg/week. There were no modifications to the MTX treatment due to the vaccination.

The patient was screened as part of an ongoing study of healthcare professionals receiving the BioNTech–Pfizer BNT162b2 COVID-19 mRNA vaccine (BNT162b2) who were included in the first vaccination group as Poland commenced its vaccination program. Each participant received two doses of the vaccine (30µg each, with an interval of 3 weeks). The study group consisted of 119 people who had not contracted COVID-19 prior to vaccination confirmed by a negative antibody test prior to the first dose of the vaccine. The mean age of the participants was 40 years old (±11.6) and 86.6% of the participants were female. The formal ethical approval was received from the Ethics Committee at the Gdansk Regional Medical Board (No KB—4/21). All participants gave written informed consent for donating their blood samples for this project.

The case patient received the first vaccine dose on 5 January 2021 and the second dose on 26 January 2021. The sole reaction after both doses was mild pain at the injection site. 

Antibody levels were assessed using an Elecsys Anti-SARS-CoV-2 S IVD assay (Roche Diagnostics, Germany) [8]. The analytical measuring interval of the assay was 0.40–250 U/mL (or up to 2500 U/mL for 10-fold, up to 25,000 for 100-fold dilution); limit of blank = 0.30 U/mL, limit of detection = 0.35 U/mL; limit of quantitation = 0.40 U/mL. All samples were frozen at −20 °C. The average antibody levels at 14 and 21 days after the first dose of vaccine for all participants were 60.6 U/mL and 121.6 U/mL, respectively, showing a two-fold increase. The participant taking MTX had negative results when tested between the first and second vaccine dose.

**Figure 1:**
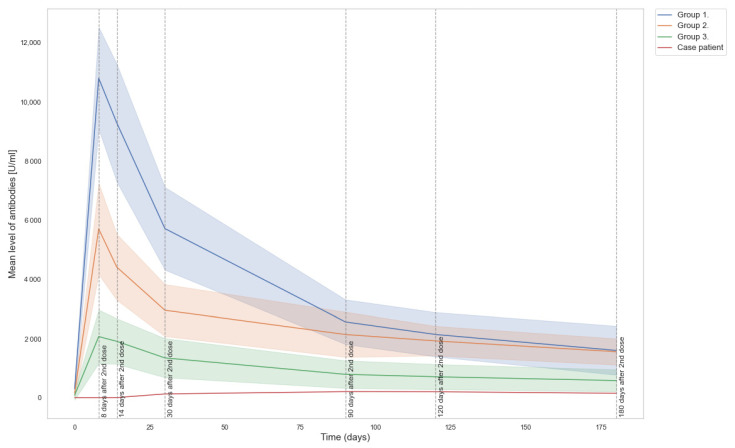
Dynamics of change in levels of SARS-CoV-2 antibodies post second dose of the vaccine (for each group mean antibody level with shading representing SD). Participants were divided into groups based on the antibody level on day 8 after the second vaccine dose: group 1 (*n* = 5) above 8000 U/mL, group 2 (*n* = 7) 4000–8000 U/mL, and group 3 (*n* = 39) below 4000 U/mL.

At the next measurement point, 8 days after the second dose, 68.1% of participants had results above 2500 U/mL which is the detection limit of the assay (after 10× dilution). The participants whose results were within the detection limit had an average antibody level of 1670.6 U/mL on day 8 after the second dose and 1319.0 U/mL on day 14, showing a 21% decrease. The case study patient had measurable antibody levels that increased from 0.595 U/mL on day 8 to 2.87 U/mL on day 14 after the second vaccine dose. At 30 days after the second dose, we saw a 35% decrease to a mean level of 854.6 U/mL in the subjects that had results within the detection limit. In the case of the MTX patient, the level of antibodies continued to increase, reaching 125.8 U/mL, then 203.7 U/mL on day 90 post second dose, remaining at a similar level (201.0 U/mL) when measured 30 days later (i.e., 120 days after the second dose). Her antibody level showed a decrease on day 180 after the second vaccine dose when it measured 147.2 U/mL. The reference group included a large number of results above the test detection limit when using the 10x dilution. While the study was ongoing, the manufacturer of the Roche assay revised the dilution recommendations. A subset of the samples (all samples for 51 participants) was reanalyzed with the 100× dilution. The reference group showed a marked decline in antibody levels between day 30 and 90 after the second dose and then leveled off from day 90 until day 180 (Figure 1).

**Figure 2:**
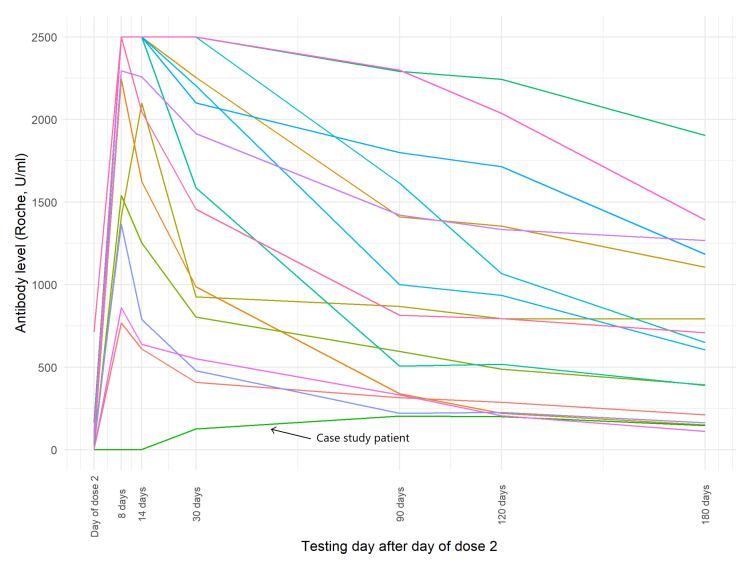
Individual dynamics of change in levels of SARS-CoV-2 antibodies post vaccination in the case study patient and 15 female study participants aged 45–55.

The individual response to vaccination can vary. Figure 2 presents the dynamics of antibodies of the case patient and 15 female participants in the age range of 45 to 55 years old without a history of SARS-CoV-2 infection. Points shown at the 2500 U/mL are measurements that exceeded the detection limit of the Roche assay, which was at the time limited to 10× dilution as per the manufacturer’s instructions.

Additionally, as we observed a negative result from the sample collected 21 days after the first dose, which we retested using a LIAISON^®^ SARS-CoV-2 TrimericS IgG assay (DiaSorin, Stillwater, OK, USA) [9]. DiaSorin showed a measurable result of 5.71 AU/mL. The subsequent samples from this patient up to day 30 after the second dose were also tested using a DiaSorin assay in addition to Roche. We also converted the results to the WHO standard units BAU/mL (binding arbitrary units/mL) according to the manufacturer’s instructions (Table 1). The additional testing using DiaSorin was performed in connection with the study comparing different serological SARS-CoV-2 assays and further details are described elsewhere [10].

## 3. Discussion

Our case study indicates that the humoral response to vaccination of a patient taking MTX can be not only lower but also delayed. Previous research on vaccination efficacy in autoimmune inflammatory rheumatic disease (AIIRD) patients has shown that MTX lowers the humoral response to some vaccines. Subesinghe S et al. [11], in their meta-analysis and systematic review concluded that MTX has a diminishing effect on response to pneumococcal vaccines but does not affect the response to influenza vaccination. To date, there are no longitudinal studies published that observe the effect of MTX on the response to the COVID-19 vaccination. 

AIIRD patients undergoing immunosuppressive therapy were not included in the COVID-19 vaccine clinical trials [12,13], so it was not known what their response to the vaccination would look like. 

Initial studies published during the COVID-19 pandemic concerning AIIRD patients, especially those using immunosuppressive therapy, described their risk of the infection, hospitalization and death. The results indicated a higher risk of mortality in people taking MTX, which seems to be important in the context of the response to vaccination in the case of our patient.

A large cohort study by Yousaf et al. [2] compared COVID-19 infection outcomes of over 32,000 patients that included 214 exposed in the previous year to tumor necrosis factor inhibitors (TNFis) or MTX. They suggest that these medications do not increase hospitalization or mortality. They found a very high mortality rate of the studied population (6.2% in people not taking TNFis or MTX—19163/31862) and a comparable 6.1% in the group exposed to TNFis or MTX (13/214). However, in the group taking MTX (128 cases, including 16 TNFis-exposed patients) the mortality rate was 9.4% (12/128). 

Additionally, similar conclusions were reached by the preliminary aggregate data of COVID-19 outcomes among autoimmune patients receiving various immunomodulatory therapies [3]. The TNFis group had no deaths (0/16), and among those receiving disease-modifying antirheumatic drugs (DMARDs) alone or with corticosteroids mortality was 21.4% (3/14).

The rollout of the vaccination programs brought forth a number of studies that started to evaluate the response to vaccination in cohorts similar to our case study patient. In general, studies of the effects of vaccination in the general population evaluate humoral response, the vaccine-induced T cellular response [14] and B cell responses [15]. All these types of response need to be taken into consideration to assess the immune response among the immunocompromised.

Serological testing is part of the evaluation of the humoral response. Another important measurement is the assessment of neutralizing activity using virus neutralization tests. However, using a live virus for such test requires a biosafety level 3 laboratory. Therefore, there is ongoing research into establishing the correlation between the results of serological immunoassays and virus neutralization tests. A study by Favresse et al. [16] showed a significant correlation between those results in the case of six different serological assays, including the Roche assay used in the study presented herein (r = 0.65, 95% CI 0.55–0.74, *p* < 0.0001).

Bugatti et al. [4] found that the humoral response tested with LIAISON SARS- CoV-2 S1/S2 IgG (DiaSorin) occurred only in 18.2% of those taking MTX with glucocorticoids and in 39.4% of those taking MTX alone who had not previously contracted COVID-19. They found that taking MTX alone carries an eight-fold higher risk of nonresponse to the first dose of BNT162b2 mRNA COVID-19 vaccine [4]. The study by Bugatti does not provide full insight into the response. Indeed, the results of antibody response after the second booster dose are not reported. In the case of our patient, there was a negative result using Roche and DiaSorin assays after the priming dose of the vaccine, but the response was positive with the DiaSorin assay following the booster dose on day 8. On day 14 after dose 2, results from both assays were positive. 

Another study of people with AIIRD was conducted in Israel by Furer et al. [5]. Here, the response to two doses of 686 people with AIIRD was compared to that of 121 people in the control group. Antibody levels were tested 2–6 weeks after the second dose of BNT162b2 vaccine. They found a vaccine response in 86% of subjects with AIIRD as opposed to 100% in the control group. IgG antibodies against S1/S2 protein were assessed by serum IgG neutralizing antibody levels against SARS-CoV-2 trimeric spike S1/S2 glycoproteins, using the LIAISON automated platform (DiaSorin). However, it appears that even though the newer version of the DiaSorin assay was used (TrimericS IgG vs. the older S1/S2 IgG), the incorrect cutoff for a positive response was used. Manufacturer’s materials for the TrimericS IgG show the value of 33.8 BAU/mL as the cutoff for determining the positive result, while the S1/S2 IgG assay has the cut off value of 15 AU/mL (as it is an older version of the assay the unit is AU/mL not BAU/mL, and to our knowledge has not been converted to BAU/mL). The determination of a sufficient response, however, could be specified based on the correlation with virus neutralization. DiaSorin shows that for the older S1/S2 IgG assay there is a high correlation of 87% with the higher microneutralization assay titer threshold of ≥ 1:160 for antibody levels over 80 AU/mL, and for the newer TrimericS IgG the correlation is 85% for the titer threshold ≥1:80 and antibody level over 520 BAU/mL. The 520 BAU/mL appears to be a better choice for determining a sufficient response to vaccination and should be measured 8 days after the second dose of the vaccine. As for normal responders, this is the peak of the humoral response [10]. Furer’s study not only appears to have used an incorrect cutoff point but also measured the antibody levels 2 to 6 weeks post-second dose. In normal responders, we have observed an average decrease of 49% between day 8 and day 30 so not standardizing the day of measurement has potentially significantly skewed the results.

An extensive study of the response of patients taking methotrexate was undertaken by Haberman et al. [6]. They studied two cohorts of patients—from New York University Langone Health with IMID, which included 51 patients with immune-mediated inflammatory diseases (IMIDs) on immunomodulatory treatment (including 25 on MTX), and from Erlangen, Germany where they studied 31 patients with IMIDs, including 20 on MTX monotherapy. Humoral response was tested in both groups after the second dose of the vaccine using different kits, none of which were based on automated, commonly used IVD systems. Both groups showed a lower percentage of patients using MTX responding to vaccination than the group not taking MTX. It was assumed that a response occurred in 72% of those taking MTX in New York and 50% in Erlangen. Cellular response was evaluated only in the New York cohort and showed a diminished response in patients taking MTX. However, several factors make data from this study difficult to interpret. It is not specified when, in relation to the second vaccine dose, the antibody measurements were performed nor whether the interval was consistent for all subjects. The use of two different ELISA kits makes it hard to interpret the comparison of the humoral response between cohorts. Additionally, as in the other discussed studies, there was only one measurement, therefore nothing can be learned about the dynamics of the response. 

Mahil et al. [7], also evaluated both humoral and cellular responses to vaccination with BNT162b2 28 days after the first dose of the vaccine. The study group included patients treated with MTX; however, their indication was psoriasis. Humoral response was evaluated with an ELISA kit and showed a diminished humoral response in the MTX group. However, in contrast to the Haberman study, they determined that cellular immunity was shown in all patients on immunosuppression, including MTX, and its level was similar to that of controls. Again, this published study result describes only the response after the first dose of the vaccine. 

A number of national rheumatology societies have issued guidelines regarding COVID-19 vaccination for AIIRD patients—the American College of Rheumatology (ACR) [17], the British Society for Rheumatology (BCR) [18], the Canadian Rheumatology Association (CRA) [19], for instance. All recommend vaccination for AIIRD patients, including those receiving MTX treatment and only ACR recommends that patients with well managed AIIRD hold off MTX for 1 week after receiving the vaccine. ACR also mentions testing antibody levels to monitor vaccine response but gives a negative recommendation for any routine testing for either assessing antibody levels or confirming prior SARS-CoV-2 infection. The BCR mentions antibody testing as an option to give patients an indication about their protection from infection in its guidelines.

AIIRD patients are part of a larger immunocompromised population. However, the group is highly heterogenous as it includes those with primary immunodeficiency, as well as secondary ones such as conditions arising from HIV, diabetes or leukemia. Medical treatment can also lead patients to become immunocompromised. This subgroup includes, for example, transplant recipients and cancer patients [20]. A study by Haidar G. et al. [21] considered a wide group of immunocompromised patients and determined wide ranging seropositivity rates after vaccination—94% in HIV patients, 80% in solid tumor or autoimmune disease patients, 55% in those with hematologic cancers and only 37% in patients who underwent solid organ transplants. The study, though yet to be peer reviewed, shows a highly heterogenous response to vaccination in this population and demonstrates a need for further research and longitudinal monitoring.

Recently, clinical trials evaluating the third vaccine dose in subsets of the immunocompromised population have been initiated. A trial of the third dose of the mRN-1273 (Moderna) vaccine in transplant recipients demonstrated increased immunogenicity in the group that received an additional vaccine dose while being shown as safe [22].

The emergence of new SARS-CoV-2 variants leads to more questions regarding the level of antibodies that would provide adequate protection from infection [23,24] and higher levels of antibodies may be required to prevent infection caused by new variants of the virus [23,24]. This makes patients on immunosuppressive therapy even more vulnerable because of their slower immune response.

## 4. Conclusions

Our study points toward the possibility of a different vaccination response model in patients undergoing immunosuppressive treatment with MTX. Our results, even though based on one case study patient, add data on the dynamics of the humoral response to what has already been published on the early response to vaccination. This appears to confirm that people with autoimmune diseases should be subject to further detailed observation during vaccinations, including testing of antibody levels, and treated as potentially at risk until the effect of vaccination is confirmed. The different available vaccines should also be tested to verify their usefulness in the case of people with autoimmune diseases and on individual immunomodulatory medications [25]. Patients who receive immunosuppressive treatment should receive the vaccine using the regular, not prolonged interval, vaccination schedule [26]. Additionally, considering a diminished humoral response and the possibility of a diminished cellular response to vaccination, especially in light of the emergence of new variants of concern of SARS-CoV-2 [23,24], along with other authors [6] we also stress the need to consider alternate vaccination strategies, such as additional vaccine doses, for MTX patients.

## Figures and Tables

**Table 1:** Roche and DiaSorin test result for samples up to day 30 after dose 2.

Day	Roche U/mL	Roche BAU/mL	DiaSorin AU/mL	DiaSorin BAU/mL
21 days after dose 1	0	0	5.71	14.846
8 days after dose 2	0.595	0.612	17.7	46.02
14 days after dose 2	2.87	2.953	34	88.4
30 days after dose 2	125.8	129.424	98.3	255.58

## Data Availability

The data presented in this study are available on request from the corresponding author.
